# The Application of Fluorescence *In Situ* Hybridization in Different Ploidy Levels Cross-Breeding of Lily

**DOI:** 10.1371/journal.pone.0126899

**Published:** 2015-05-26

**Authors:** Qing Wang, Jingmao Wang, Yiying Zhang, Yue Zhang, Shunchao Xu, Yingmin Lu

**Affiliations:** College of Landscape Architecture, Beijing Forestry University, Beijing, 100083, China; Brunel University, UNITED KINGDOM

## Abstract

21 crossing were conducted between Asiatic Lily with different ploidy levels, the results showed that the interploidy hybridization between diploid and tetraploid lilies was not as successful as intraploidy hybridization. Regardless of male sterility, triploid lilies could be used as female parents in the hybridization which the progenies were aneuploidy. 3x×4x crosses could be cultured more successfully than 3x×2x crosses. 45S rDNA was mapped on the chromosomes of seven *Lilium* species and their progenies using fluorescence *in situ* hybridization (FISH). FISH revealed six to sixteen 45S rDNA gene loci, and normally the sites were not in pairs. The asymmetry indexes of LA (Longiflorum hybrids × Asiatic hybrids) hybrids was higher than Asiatic hybrids, the evolution degree was LA hybrids > Asiatic hybrids. 45S rDNA distributed variably on chromosome 1-10 and 12 among Asiatic hybrids. Chromosome 1 had invariable sites of 45S rDNA in all Asiatic hybrids, which could be considered as the characteristic of Asiatic hybrids. LA hybrid ‘Freya’ had two sites of 45S rDNA on one homologous chromosome 5, and also it could be found in the progenies. The karyotype and fluorescence *in situ* hybridization with 45S rDNA as probe were applied to identify the different genotypes of 9 hybrids. Typical chromosomes with parental signal sites could be observed in all the genotypes of hybrids, it was confirmed that all the hybrids were true.

## Introduction

The genus *Lilium*, which belongs to the family Liliaceae, is composed of approximately 100 species and is widely distributed across temperate regions of Asia, Europe, and North America [1, 2]. Most species of the Sinomartagon, such as *L*. *dauricum*, *L*. *maculatum*, *L*. *concolor*, *L*. *leichtlinii*, *L*. *davidii* and *L*. *cernuum*, are distributed in East Asia.

Chromosome morphology of a majority of *Lilium* species has been confirmed [3]. All the species of the genus *Lilium* are diploid (2n = 2x = 24) [3] except for *Lilium lancifolium* in that triploids (2n = 3x = 36) occur as well [4]. In addition, cytological investigation by C-banding has been applied in many different *Lilium* species [5, 6, 7, 8]. Karyotype analysis of Chinense wild *Lilium* species has also been done by C-banding, including *L*. *speciosu*, *L*. *pumilum*, *L*. *concolor*, *L*. *rosthornii*, *L*. *davidii*, *L*. *jinfushanense*, *L*. *regale*, *L*. *dahuricum* and *L*. *sargentiae*. C-banding has been widely used for chromosome analysis for about 44 years [9, 10]. Lim [11] analyzed root tip chromosomes *of L*. longiflorum and red *L*. *rubellum* by Giemsa C—zoning, but only eleven shallow belts were observed. Marasek and Orlikowska [12] applied Giemsa C—zoning technology to the identification of the lily hybrid family. However, many lily chromosome length and arm ratio did not have so many differences, and the source of lily hybrid progeny chromosome and the identification of chromosome translocation cannot be judged by karyotype analysis. Besides, *Lilium* chromosomes are relatively large [3] and it is difficult to represent genomes of this size with C-banded DNA [8]. Fluorescence in situ hybridization (FISH), which maps repetitive or single-copy sequences on the chromosomes, now complements banding technologies, along with the use of DNA-specific fluorochromes, which elucidate local variation in DNA and (or) chromatin composition. The most common application of FISH is the localization of rDNA families on the chromosomes. FISH of 45S and 5S rDNAs have been widely used in various ornamental flowers and plants including *Hordeum* [13], *Brassica* [14, 15], *Cucumis* [16], *Nicotiana* [17] and *Tulipa* [18]. The wild *Lillium* species were classified as three categories according to the characteristics of the 45S rDNA and 5S rDNAs signal loci [19]. Hwang [20] detected five pairs of 45S rDNA signals and a pair of 5S rDNA signals in both diploid and triploid *L*. *lancifolium*. 45S rDNA are usually distributed on the chromosome in the form of series and 45S rDNA numbers and distribution of different species are different, therefore we can apply 45S rDNA into the study of phylogenetic relationship, genetic relationship and karyotype.

Cross breeding is a major means of lily breeding and hybrid affinity is the prerequisite for success. The rich ploidy of parents has certain influence on hybridization affinity. Therefore, the research of ploidy can guide breeding purposely, shorten the breeding period and reduce the blindness of breeding. The aims of the study were to research the relationship between lily crossbreeding affinity and ploidy level, to identify the hybrids, to explore the distribution of 45S rDNA on the chromosomes in Asiatic lily. The ploidy level was investigated and interploid crossing was made by conventional pollination technique. Furthermore, the crossbreeding affinity was evaluated. The karyotype and fluorescence *in situ* hybridization with 45S rDNA probe were applied.

## Materials and Methods

### Plant Materials

Asiatic hybrid cultivars ‘Renoir’, ‘Gironde’, ‘Navona’, ‘Loreto’, ‘Detroit’ and ‘Tresor’ and ‘Freya’ (Longifolium lily×Asiatic lily) were imported from the Netherlands. The progenies of ‘Renoir’ × ‘Gironde’, ‘Gironde’ × ‘Renoir’, ‘Gironde’ × ‘Tresor’, ‘Loreto’× ‘Detroit’, ‘Navona’ × ‘Loreto’, ‘Navona’ × ‘Tresor’, ‘Navona’ × ‘Detroit’, ‘Freya’ × ‘Detroit’ and ‘Freya’ × ‘Loreto’.

### Hybridization pollination and embryo rescue

In order to prevent self-pollination, stamens of female parent were removed in the bud stage. After flowering, pollination were carried out at 8:00 ~ 10:00 am and promptly bagged to avoid other pollen contamination. Embryo Rescue was conducted when the fruits became soft and yellow. The fruits were washed and disinfected with 75% alcohol and 1% NaClO in clean bench. Then the seeds were inoculated onto the culture medium after removing seed coat and cultured in a conventional culture chamber.

### Chromosome Preparation

Root tips from pot-grown plants were pretreated with 0.7 mmol/L cycloheximide solution for 4~6 h at room temperature and fixed in glacial acetic acid: ethanol (1:3, v/v) for 24 h. Root tips were thoroughly washed and then macerated using an enzyme cocktail (1% cellulase, and 1% pectolyase) at 37°C for 1 h, followed by rinsing in distilled water. Squash preparations were made in a drop of 45% acetic acid. The microscope slides were frozen in liquid nitrogen and the cover slips removed with a razor blade. Slides were then finally dehydrated in absolute ethanol, air-dried, and stored at—20°C in a freezer until used.

### Fluorescence *in Situ* Hybridization

FISH was performed according to the technique described by [21]. The slides were pre-treated with 200μl RNase A (DNase-free, 100 μg/mL) for 1 hour at 37°C and washed in 2x SSC three times and then post fixed in 4% (w/v) para-formaldehyde solution for 10 min and then dehydrated using an ethanol gradient (70%, 95% and 100%) at -20°C for 3 min each. The hybridization mixture containing 50% deionized formamide (v/v), 10% dextrin sulfate(w/v), 2x SSC 0.25% SDS and 1μl probe DNA was then denatured at 70°C for 10 min and immediately chilled on ice for 5 min; 40 μl of the probe mixture was applied to the denatured chromosomal DNA and covered with a glass cover slip. Slides were then placed in a humid chamber at 37°C overnight. After hybridization, a glass cover slip was removed and the slides were immersed in 2x SSC for 15 min and then washed in 0.1x SSC at 42°C for 30 min and then immersed in 1x PBS for 5 min and added 200 μl 1x PBS solution (containing 1% blocking reagent) and covered plastic film for 5 min and added 100 μl blocking buffer (containing 10 μg/mL anti-Dig-Rhoda mine) and covered plastic film for 5 min. Then remove the plastic film and immerse the slides for 3 times, 5 min each. Finally added 100 μl 6-diamidino-2-phenylindole (1 μg/mL DAPI) and covered plastic film for 5 min and added 7μl sealing liquid resistant to fluorescence quenching after removed the film and covered with a glass cover slip. The slides were examined under the Nikon BX 61 fluorescent microscope. Putative homologous chromosomes were then confirmed based on their morphological characteristics, FISH and DAPI bands results. At least ten cells showing well-spread metaphase chromosomes were used in karyotype analysis. The individual chromosome length was measured by software and determined chromosome number on the basis of short arm length order according to Lim et al. [21]. Chromosome types were classified according to arm ratio value by Levan et al. [22].

## Results and Discussion

### The relationship between lily hybridization affinity and the chromosome ploidy

Triploid plants had many excellent horticultural traits and more and more attentions were payed to it. At present, the homologous triploid and allotriploid had been successfully applied in cross breeding of many plant species [23–26]. Unlike other triploid plants such as seedless watermelon and banana, mostly triploid lily were male sterility, but can be used as female parent and hybrid with appropriate male parent, this was because the embryo sac is fritillaria type embryo sac (fritillaria type embryo sac) [27]. The hybrid progenies of 5 hybrid groups (‘Navona’× ‘Loreto’, ‘Navona’× ‘Tresor’, ‘Navona’ ×‘Detroit’, ‘Freya’× ‘Detroit’ and ‘Freya’× ‘Loreto’) which triploid variety used as female parent were successfully obtained. According to the theory of the formation of megaspore embryo sac in fritillaria type plant [29], we can deduce the endosperm ploidy of 3x × 2x hybrid progeny is 7x while the endosperm ploidy of 3x×4x is 8x. The endosperm level was a major cause of seed development or abortion; therefore, the survival of aneuploid embryo (3x × 2x / 4x) depended on the endosperm euploidy [30]. Zhou etc. [31] hypothesized the hybrid endosperm (triploid lily as female parent) with at least five same genome was the necessary condition to obtain hybrid progenies. This hypothesis could explain whether 3x × 2x / 4x hybrid succeeded or not and was of great significance for lily breeding. The incompatibility was caused by more different parental gender composition; the number of chromosome in parents had great influence on cross incompatibilities. Johnston [32] proposed EBN (endosperm balance number) hypothesis which clarified the relationship between ploidy and cross-compatibility, only when the genetic composition ratio of endosperm of parents was 1:2, endosperm could develop normally and produce seeds, this hypothesis could explain whether the interspecific hybridization and multiple sexual hybridization woule be successful or not [33].

The seed setting rate of sexual hybridization between diploid and tetraploid (interploidy hybridization) was low. There was a certain degree of incompatibility compared with intraploidy hybridization, which was due to the changes in the ratio of genome composition on embryo and endosperm of the the parents [23]. The ratio of genome composition on endosperm of 2x × 4x and 4x × 2x was respectively 1:1 and 1:4 and endosperm development was incomplete, so it was difficult to produce seeds and hybrid seed setting rate was low. In this study, cross compatibilities between diploid and tetraploid (interploidy hybridization) were poorer (Table 1), only a group received triploid hybrid progenies and the results of reciprocal cross were different. The triploid lily as the female parent in hybrid could foster aneuploid progenies, provide rich material for lily breeding and the hybrid of 3x × 4x is easier than that of 3x × 2x which were in conformity with the previous results [30, 31]. The larger number of chromosome from male parent might promot the development of endosperm [34, 35].

**Table 1:** The results of crosses.

Cross	Mean fruit rate (%)	Mean embryo rate (%)	Cross	Mean fruit rate (%)	Mean embryo rate (%)
2x×2x	80	10	2x×3x	0	0
4x×4x	88.3	2.1	4x×3x	5	0
2x×4x	40	1.7	3x×2x	77.5	1.1
4x×2x	58.3	0	3x×4x	93.3	8.3

In some plants, hybrid progeny of diploid and tetraploid was tetraploid, such as blueberries [36], which was caused by the production of somatic gametes (2n gametes) during meiosis anomalies in diploid plant [37]. Watanabe [38] obtained tetraploid potato through the hybridization of diploid potato and tetraploid hybrids. The progenies could survive when triploid female parent provided euploid gametes, and in most other plants, the progenies of 3x × 2x hybrid were usually diploid while the progenies of 3x × 4x hybrid were usually tetraploid [25, 32]. But 3x × 2x /4x hybrid lily progeny was usually aneuploidy [39–41]. In this study, progeny was aneuploidy (x+6) when female parent was triploid, this can also be explained by the theory of the formation of megaspore embryo sac in fritillaria type plant [27]. The euploid or aneuploid with different chromosome number could be obtained through the hybridization of different ploidy lily, resulting in a rich variation; these variations can be conserved by scale cutting or organizational culture.

### The Karyotype Diversity of Seven *Lilium* Cultivars

**Fig 1:**
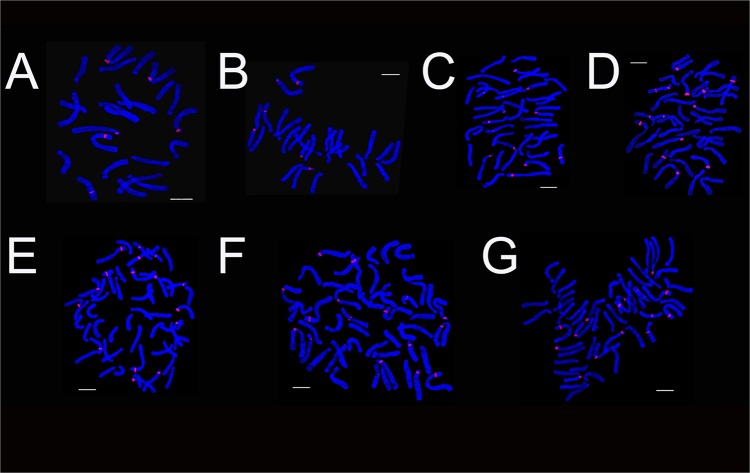
The karyotype of *Lilium* cultivars using 45S rDNA (red) probes. Asiatic cultivar ‘Renoir’ (A), ‘Gironde’(B), ‘Navona’ (C), ‘Detroit’ (E), ‘Loreto’ (F), ‘Tresor’ (G) and LA cultivar ‘Freya’ (D). Bar = 10 um.

**Fig 2:**
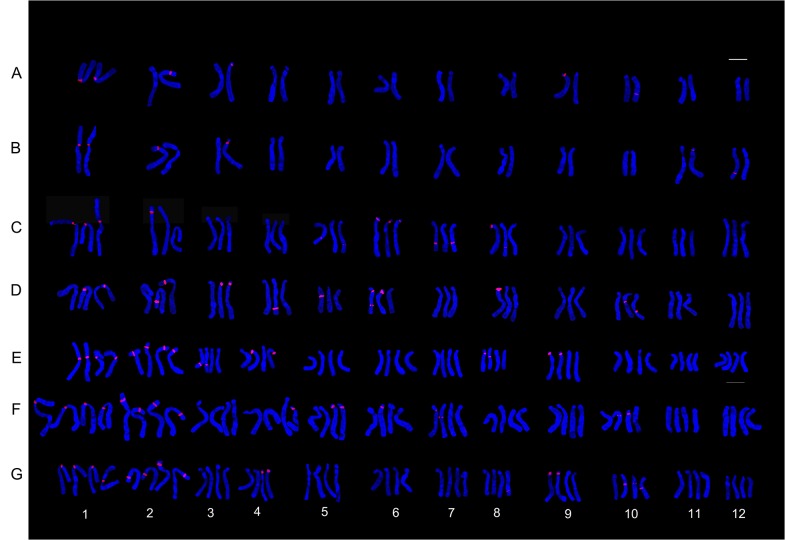
FISH karyotype of *Lilium* cultivars using 45S rDNA (red) probes. Asiatic cultivar ‘Renoir’ (A), ‘Gironde’(B), ‘Navona’ (C), ‘Detroit’ (E), ‘Loreto’ (F), ‘Tresor’ (G) and LA cultivar ‘Freya’ (D). Bar = 10 um.

Most of the wild lilies are diploid and the ploidies of lily varieties are abundant. Most of the intra-group hybrids (Asiatic hybrids, the Oriental hybrids and Longiflorum hybrids, etc.) are diploid whereas the majority of inter-group hybrids (LA-Longiflorum hybrids × Asiatic hybrids, LO-Longiflorum hybrids × Oriental hybridsand OT-Oriental hybrids × Trumpet hybrids, etc.) are triploid. Asiatic hybrids were mainly diploid and some were triploid, tetraploid and aneuploid [28]. The number of chromosomes in six Asiatic hybrids (Figs 1 and 2) were observed 2n = 2x = 24 (‘Renoir’, ‘Gironde’), 2n = 3x = 36 (‘Navona’) and 2n = 4x = 48 (‘Detroit’ ‘Loreto’ ‘Tresor’). The LA hybrid ‘Freya’ was triploid (2n = 3x = 36).

The karyotype characteristics of Asiatic lily varieties were different from the karyotype characteristics of LA lily varieties (Table 2). The chromosome of the *Lillium* belongs to large chromosome and karyotype is relatively stable. Generally, lily chromosomes are composed of two large metacentrics or submetacentrics and ten subtelocentrics or telocentrics [3] Two pairs of metacentrics (chromosomes 1, 2), nine pairs of subtelocentrics (chromosomes 3–11)and a pair of telocentrics (chromosomes 12) were found in ‘Loreto’, a pair of metacentrics, a pair of submetacentrics and nine pairs of subtelocentrics belonged to ‘Tresor’ and a pair of metacentrics (chromosomes 1), a pair of subtelocentrics (chromosomes 2), nine pairs of subtelocentrics (chromosomes 3–11)and a pair of telocentrics (chromosomes 12) were observed in ‘Navona’. ‘Detroit’, ‘Renoir and ‘Gironde’ consisted of two metacentrics (chromosomes 1, 2) and ten subtelocentrics (chromosomes 3–12). However, LA hybrid ‘Freya’ was composed of a pair of metacentrics (chromosomes 1), a pair of submetacentrics(chromosomes 2), six pairs of subtelocentrics(chromosomes 3, 4, 5, 6, 7, 10) and four pairs of telocentrics(chromosomes 8, 9, 11 and 12). Asiatic cultivars were different from LA cultivars in the karyotype.

**Table 2:** Summary of karyotype and morphological data of seven cultivars.

Cultivar	Karyotype formulate	Average arm ratio	Karyotype type	As.K^a^/%	45S rDNA	
					Number	No.
‘Renoir’	2n = 2x = 24 = 4m+20st	4.23	3A	76.75	6	1,2,3,9,10
‘Gironde’	2n = 2x = 24 = 4m+20st	4.57	3A	78.35	6	1,2,3,7,12
‘Navona’	2n = 3x = 36 = 3m+3sm+27st+3t	4.96	3B	79.01	13	1,2,5,6,7,8
‘Detroit’	2n = 4x = 48 = 8m+40st	4.34	3B	77.79	16	1,2,3,4,8,9
‘Tresor’	2n = 4x = 48 = 4m+4sm+40st	4.08	3B	77.43	16	1,2,4,8,9,10
‘Loreto’	2n = 4x = 48 = 8m+36st+4t	4.79	3B	78.23	16	1,2,4,5,6,7,10
‘Freya’	2n = 3x = 36 = 3m+3sm+18st+12t	6.12	3A	81.61	14	1,2,3,4,5,6,8,10

Note

^a^As.K means asymmetric coefficients

The range of mean arm ratio of Asiatic lily varieties was small, ranging from 4.08 (‘Tresor’) to 4.96 (‘Navona’) while the mean arm ratio of LA species ‘Freya’ was 6.12, significantly higher than the Asiatic lily varieties. The asymmetrical coefficient of the *Lillium* are approximately 80%, belongs to the highly asymmetric type. According to the point of karyotype evolutionary by Stebbins that evolution occurred from symmetry to asymmetry [42], thus the degree of the evolution in *Lillium* is higher. The asymmetry coefficient of seven cultivars ranged 76.29% ~ 81.68% and LA variety ‘Freya’ had the highest asymmetry coefficient so that ‘Freya’ is relatively primitive.

### The Diversity of Fluorescence in Situ Hybridization

**Fig 3:**
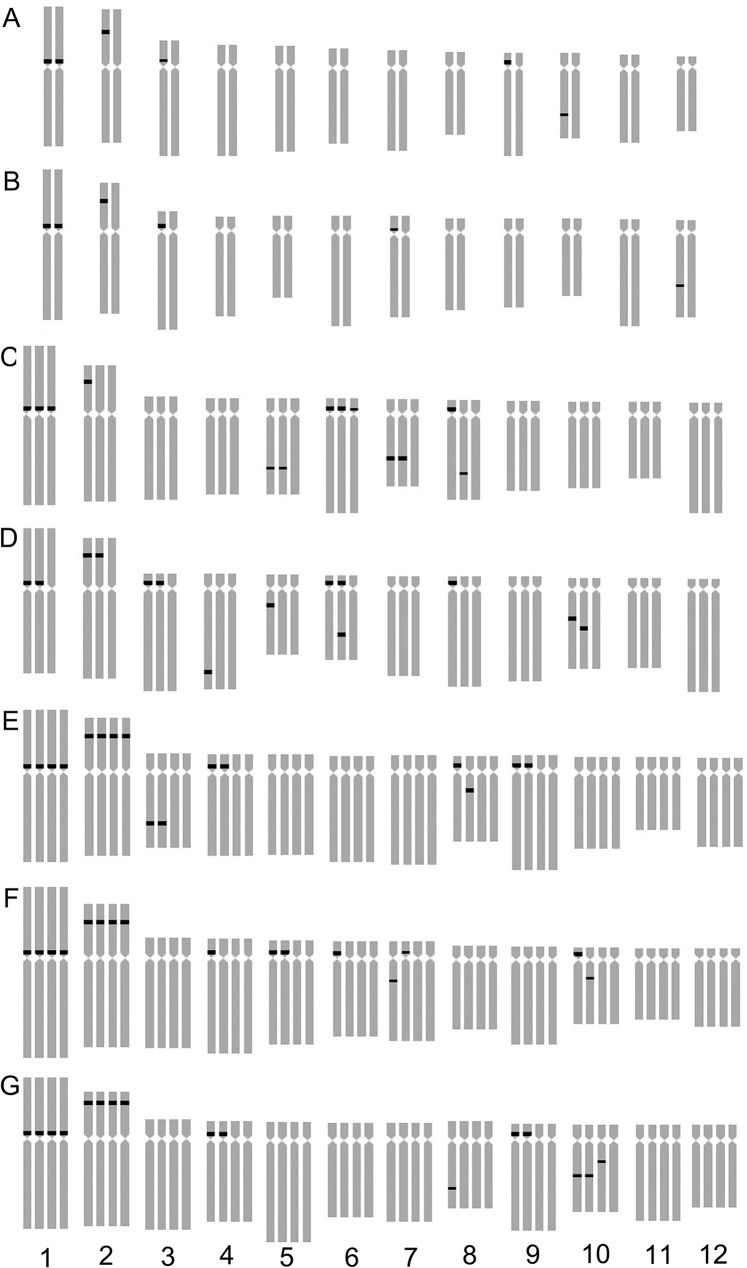
Ideograms showing the distribution of 45S rDNA on the chromosomes in *Lilium* cultivars. Asiatic cultivar ‘Renoir’ (A), ‘Gironde’(B), ‘Navona’ (C), ‘Detroit’ (E), ‘Loreto’ (F), ‘Tresor’ (G) and LA cultivar ‘Freya’ (D). Bar = 10 um.

The karyotype of the *Lillium* was similar, but the number and distribution of 45S rDNA signal were different in different species or cultivars. ‘Renoir’ and ‘Gironde’ both had six 45S rDNA signal loci respectively in chromosomes 1, 2, 3, 9, 10 and 1, 2, 3, 7, 12(Figs 1, 2 and 3). ‘Navona’ and ‘Freya’ respectively had thirteen (chromosomes 1, 2, 5, 6, 7, 8) and fourteen (chromosomes 1, 2, 3, 4, 5, 6, 8, 10) 45S rDNA signal loci (Figs 1, 2, 3C and 3D). ‘Detroit’, ‘Tresor’ and ‘Loreto’ had sixteen 45S rDNA signal loci (Figs 1, 2, 3E, 3F and 3G). The number of the 45S rDNA signal loci increased with the increase of chromosome ploidy. Cultivars had a close relationship with its original group. Asiatic hybrid lily was got by intra-group cross of Sinomartagon. Fluorescence in situ hybridization (FISH) were carried out to elucidate inter-specific relationships among wild *Lilium* species distributed in Korea, the results domenstrated that diploid *L*. *lancifolium*, triploid *L*. *lancifolium*, *L*. *maximowiczii*, *L*. *cemuum*, *L*. *callosum*, *L*. *concolor* var. *patheneion*, *L*. *concolor* var. *vuschianum* and *L*. *dauricum* had two pairs of 45S rDNA gene loci on the short arm of chromosomes 1 and 2, *L*. *amabile* only had a pair of 45S rDNA gene loci on the short arm of chromosomes 1. Asiatic cultivars had the same 45S rDNA gene loci on the short arm of chromosomes 1 with the wide species of Sinomartagon. This clarified chromosome 1 was very stable and can be used as the characteristics of the Asiatic lily (Fig 3). ‘Loreto’, ‘Detroit’ and ‘Tresor’ had a pair of 45S rDNA gene loci on chromosome 2 so we speculated that the parents of the three cultivars all had a pair of 45S rDNA gene loci on chromosome 2. However, only one 45S rDNA signal were located on chromosome 2 in ‘Renoir’, ‘Gironde’ and ‘Navona’, we concluded that one of the parents of the three cultivars had 45S rDNA signal on chromosome 2 and another did not have. The laws of distribution of 45S rDNA signal loci on chromosome 3–12 were not obvious in *Lillium* species. The 45S rDNA signal loci can be found on different chromosomes except for chromosome 11 among different genotypes.

45S rDNA signal loci occurred in pairs in most of *Lillium* species but usually did not appear in pairs in lily hybrids. Lily hybrids are obtained by hybridization of different species and various signal loci result in single appearance of 45S rDNA signal loci. The distribution of 45S rDNA signal loci were different between Asiatic hybrids and Longiflorum hybrids and LA hybrid ‘Freya’ just had two 45S rDNA signal loci on chromosome 1, therefore, we concluded that 45S rDNA signal loci were not located on chromosome 1 of Longiflorum hybrids. Two 45S rDNA signal loci were found on the same chromosome of chromosome 11 in ‘Freya’ which might be caused by gene translocation or inversion during the evolution or the exchange and restructuring between homologous chromosomes during meiosis [43].

In *Lilium* species, 45S rDNA gene loci located almost all chromosomes except chromosomes 11. This observation was not consistent with previous studies in European mountain *Lilium* [44] and *Lilium henryi* [45].The 45S rDNA signal loci of *Lillium* species and cultivars numerously distributed in the regions which located close to centromere in chromosomes of short arms and a few positioned in the regions of long arms. Moreover, the 45S rDNA signal loci were mostly located in the regions which located close to centromere, such as Oriental cultivars ‘Marco Polo’ and ‘Expression’ [45] while chromosome 3 carried the only pair of 5S rDNA gene loci.

### The Hybrid Identification of 9 Hybrid Progenies by FISH

The chromosomes from the female parent and male parent could be detected in every hybrid progeny so that the hybrids were real. The hybrids of ‘Renoir’× ‘Gironde’ and ‘Gironde’ ×‘Renoir’ were diploid and had two metacentrics and ten subtelocentrics. The karyotype was 3A, the average arm ratio and karyotype asymmetry coefficient was close to parents (Table 3).

**Table 3:** The karyotypes and morphological data of the progenies.

Genotype	Karyotype formulate	Average arm ratio	Karyotype type	As.K^a^/%	45S rDNA		
					Number	From female	From male
RG1	2n = 2x = 24 = 4m+20st	4.96	3A	79.01	7	1,2,9	1,2,3,12
RG2	2n = 2x = 24 = 4m+20st	4.34	3A	77.79	6	1,3	1,2,3,12
RG3	2n = 2x = 24 = 4m+20st	4.08	3A	77.43	5	1,9,10	1,2
GR1	2n = 2x = 24 = 4m+20st	4.56	3A	77.89	6	1,2	1,3,9,10
GR2	2n = 2x = 24 = 4m+20st	4.24	3A	76.80	6	1,2,3	1,3,10
GT1	2n = 3x = 36 = 3m+3sm+30st	4.51	3B	78.19	12	1,2,7,12	1,2,9,10
GT2	2n = 3x = 36 = 3m+3sm+30st	4.07	3B	76.29	13	1,2,3,12	1,2,9,10
LD1	2n = 4x = 48 = 8m+36st+4t	4.51	3B	79.44	14	1,2,5,6,10	1,2,9
LD2	2n = 4x = 48 = 8m+40st	4.07	3B	78.90	15	1,2,4,5,10	1,2,9
NL1	2n = 4x-1 = 47 = 4m+4sm+31st+4t	4.83	3B	79.11	16	1,5,6,7,8	1,2,4,7,10
NL2	2n = 4x-6 = 42 = 8m+34st	4.43	3B	77.46	14	1,5,6,7,8	1,2,7, 10
NL3	2n = 4x-4 = 44 = 3m+4sm+37st	4.26	3B	77.65	13	1,2,6,7,8	1,2,4,5,10
NL4	2n = 4x-6 = 42 = 8m+34st	4.78	3B	78.66	16	1,2,6,7,8	1,2,5,6,7,10
NT1	2n = 3x = 36 = 3m+3sm+30st	4.92	3A	79.54	11	1,6,7	1,2,8,9,10
NT2	2n = 3x+4 = 40 = 3m+4sm+33st	4.92	3B	76.69	12	1,5,6,8	1,2,8,9,10
NT3	2n = 3x+6 = 42 = 7m+35st	5.26	3B	79.37	13	1,5,6,8	1,2,8,9,10
ND1	2n = 3x+2 = 38 = 6m+32st	4.80	3A	78.56	12	1,6,7,8	1,2,3,4,9
ND2	2n = 3x+4 = 40 = 4m+3sm+33st	4.80	3B	79.24	15	1,2,5,6,7	1,2,4,9
ND3	2n = 3x+1 = 37 = 6m+28st+3t	4.87	3B	78.75	13	1,2,6,7, 8	1,2,4,8,9
ND4	2n = 4x-1 = 47 = 7m+40st	4.55	3B	78.34	15	1,2,5,6,7,8	1,2,4
FD1	2n = 5x = 60 = 10m+50st	5.24	3B	79.35	23	1,2,3,4,5,6,8,10	1,2,3,4,8
FD2	2n = 5x-5 = 55 = 8m+42st+5t	5.36	3B	79.58	20	1,2,3,4,5,6,8,10	1,2,3,4,8
FD3	2n = 5x-2 = 58 = 4m+4sm+35st+15t	5.47	3B	80.75	20	1,2,3,4,5,6,8,10	1,2,4,8
FL1	2n = 3x+5 = 41 = 4m+4sm+22st+11t	5.37	3B	79.81	17	1,2,3,6,10	1,2,5,6,7,10
FL2	2n = 3x+4 = 40 = 8m+29st+3t	5.02	3A	79.41	17	1,2,5,6,8,10	1,2,7,10
FL3	2n = 5x-3 = 57 = 8m+39st+10t	4.99	3B	79.17	21	1,2,3,5,6,8,10	1,2,5,6,7,10

Note

^a^ As.K means asymmetric coefficients

**Fig 4:**
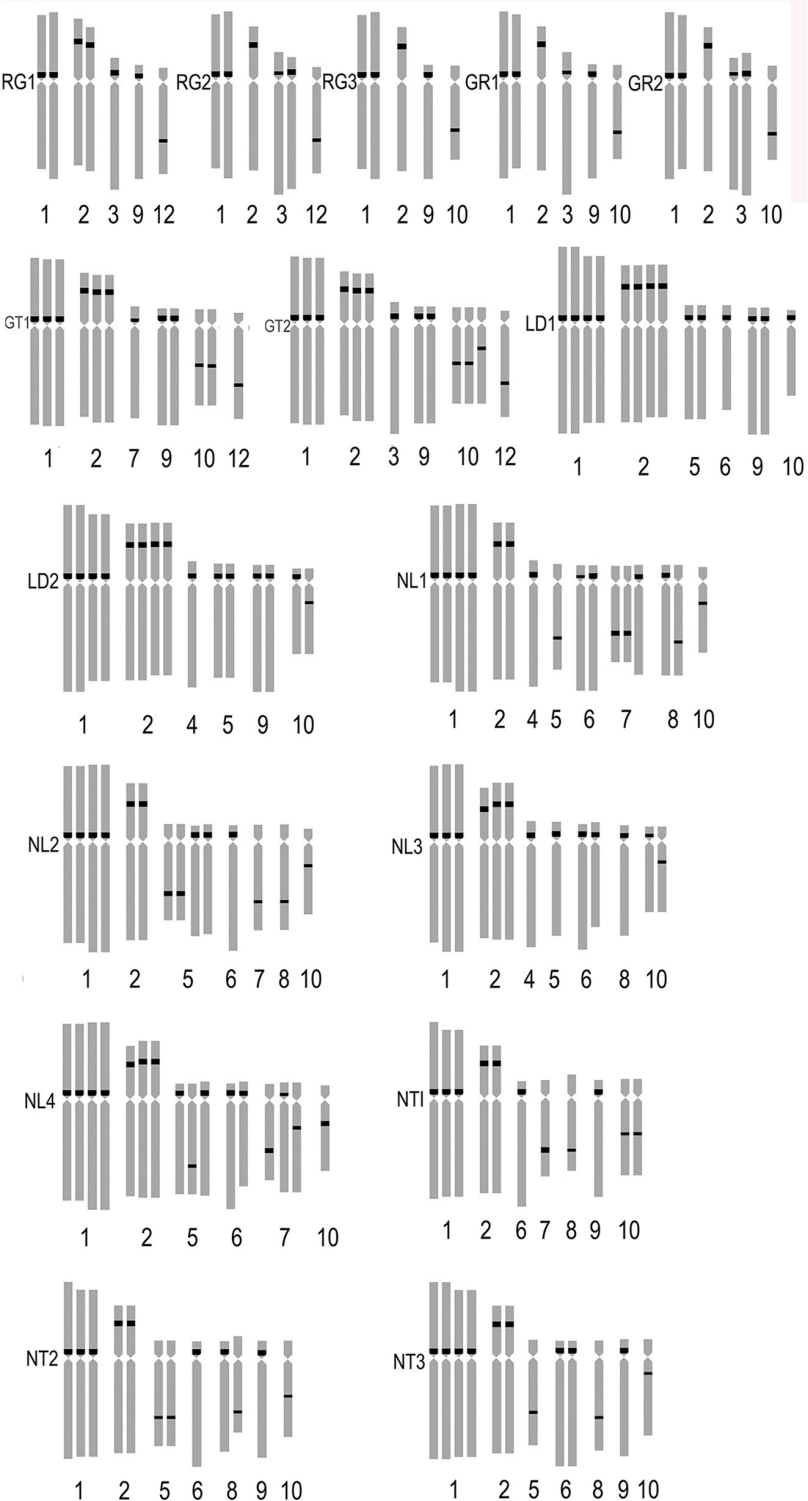
Ideograms showing the distribution of 45S rDNA on the chromosomes in the hybrid progenies (RG, GR, GT, LD, NL, NT). ‘Renoir’ ×‘Gironde’, ‘Gironde’× ‘Renoir’, ‘Gironde’× ‘Tresor’, ‘Loreto’× ‘Detroit’, ‘Navona’× ‘Loreto’, ‘Navona’× ‘Tresor’.

**Fig 5:**
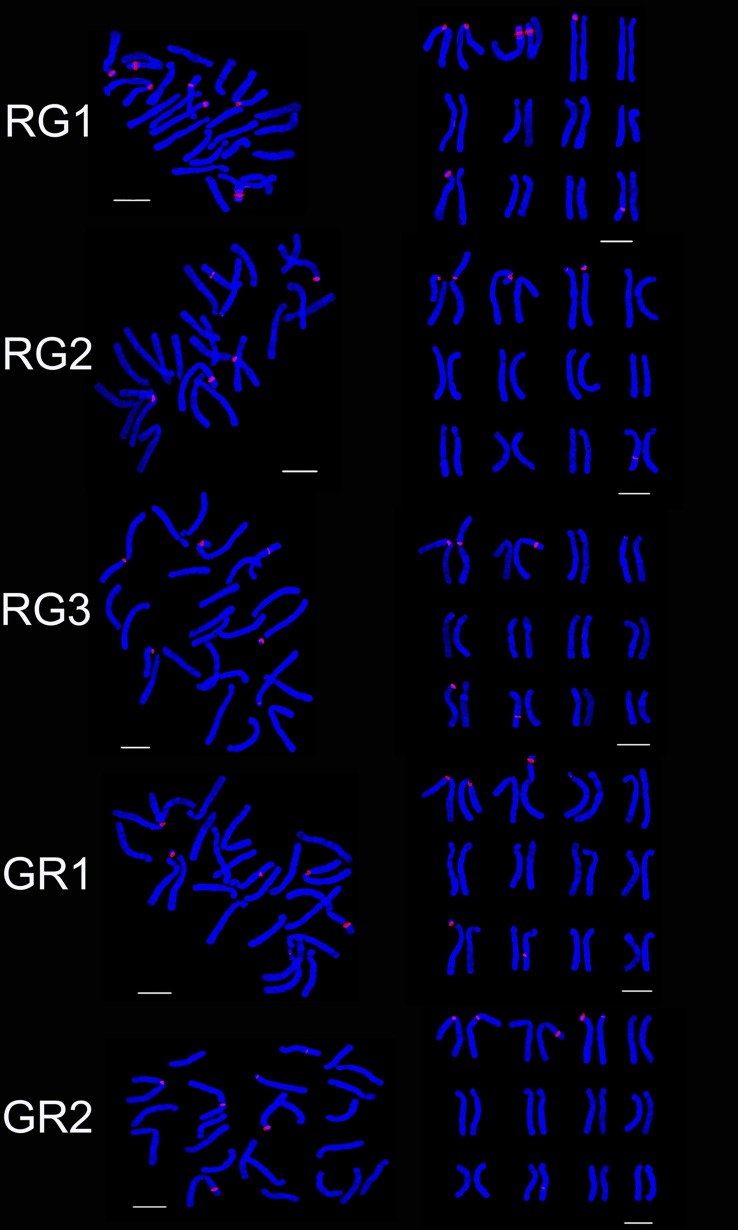
FISH karyotype of the hybrid progenies (RG1, RG2, RG3; GR1, GR2) using 45S rDNA probes. RG1, RG2, RG3 are the progenies of ‘Renoir’ ×‘Gironde’; GR1, GR2 are the progenies of ‘Gironde’× ‘Renoir’. Bar = 10 um.

FISH showed that the three hybrid progeny (RG) had same chromosomes, but the number and distribution of 45S rDNA signal were different. The number of 45S rDNA signal loci of RG1, RG2 and RG3 were respectively 7, 6 and 5. On chromosome 2, RG1 had two signal loci while RG2 and RG3 had one signal loci. On chromosome 3, RG1 had one signal while RG2 had 2 signals. One signal loci was located on the chromosome 9 of RG1 and RG3, on the chromosome 8 of RG1 and RG2 and on the chromosome 10 of RG3. The 45S rDNA signal loci were located on chromosomes 1 and 2 of the parents and the progeny (RG) (Figs 3, 4 and 5). The hybrid progeny (GR) also had the same chromosome. GR1 had one signal on chromosome 3 where GR2 had two signals. Parents and progeny both had 45S rDNA signal loci on chromosome 1, 2 and 3 (Figs 3, 4 and 5).

**Fig 6:**
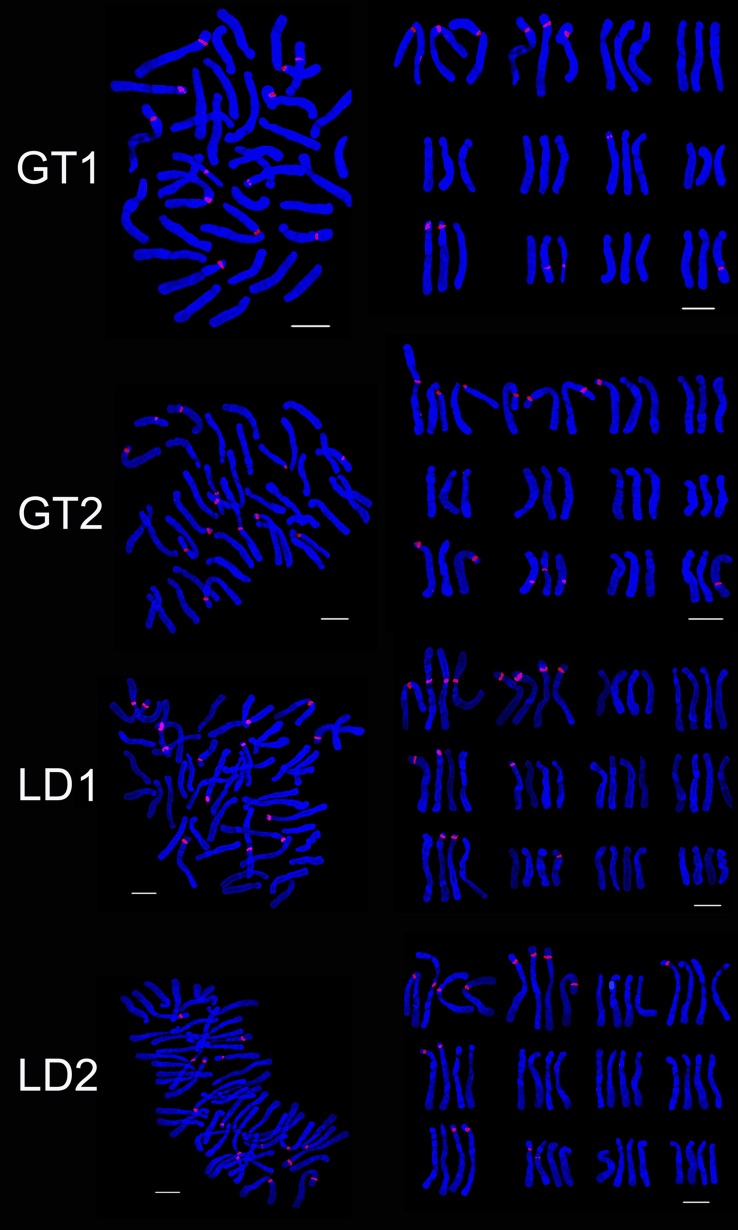
FISH karyotype of the hybrid progenies (GT1, GT2, GT3; LD1, LD2) using 45S rDNA probes. GT1, GT2, GT3 are the progenies of ‘Gironde’× ‘Tresor’; LD1, LD2 are the progenies of ‘Loreto’× ‘Detroit’. Bar = 10 um.

The hybrids of ‘Gironde’ × ‘Tresor’ were triploid and the karyotype were 3B the same as the male parent. Chromosomes of the progeny were same with male parent ‘Tresor’ which were composed of one metacentrics, one submetacentrics and ten subtelocentrics. The average arm ratio and asymmetrical coefficient of GT1 were close to female parent whie that of GT2 were close to male parent (Table 3). The number of 45S rDNA signal loci in GT1 and GT2 were 12 and 13. One signal loci were observed on chromosome 3 of GT2 and chromosome 7 of GT1. GT1 and GT2 respectively had 2 and 3 signal loci on chromosome 10. The 45S rDNA signal loci were located on chromosomes 1 and 2 of the parents (Figs 4 and 6).

The two hybrids (LD) had 48 chromosomes, which were tetraploid. The karyotype was 3B. LD1 had the same karyotype with the female parent while the karyotype of LD2 is similar to the male parent. The average arm ratio of LD1 was between parents while that of LD2 was less than the parent. The asymmetry coefficient of LD1 and LD2 were higher than parents (Table 3). The 45S rDNA signal loci of LD1 and LD2 were 14 and 15 respectively. On chromosome 9, LD1 and LD2 had two signal loci and LD1 had one signal loci on chromosome 6. One and two signal loci were respectively positioned on chromosome 10 of LD1 and LD2. The 45S rDNA signal loci were positioned on chromosomes 1, 2, 3 and 11 of the parents (Figs 4 and 6).

**Fig 7:**
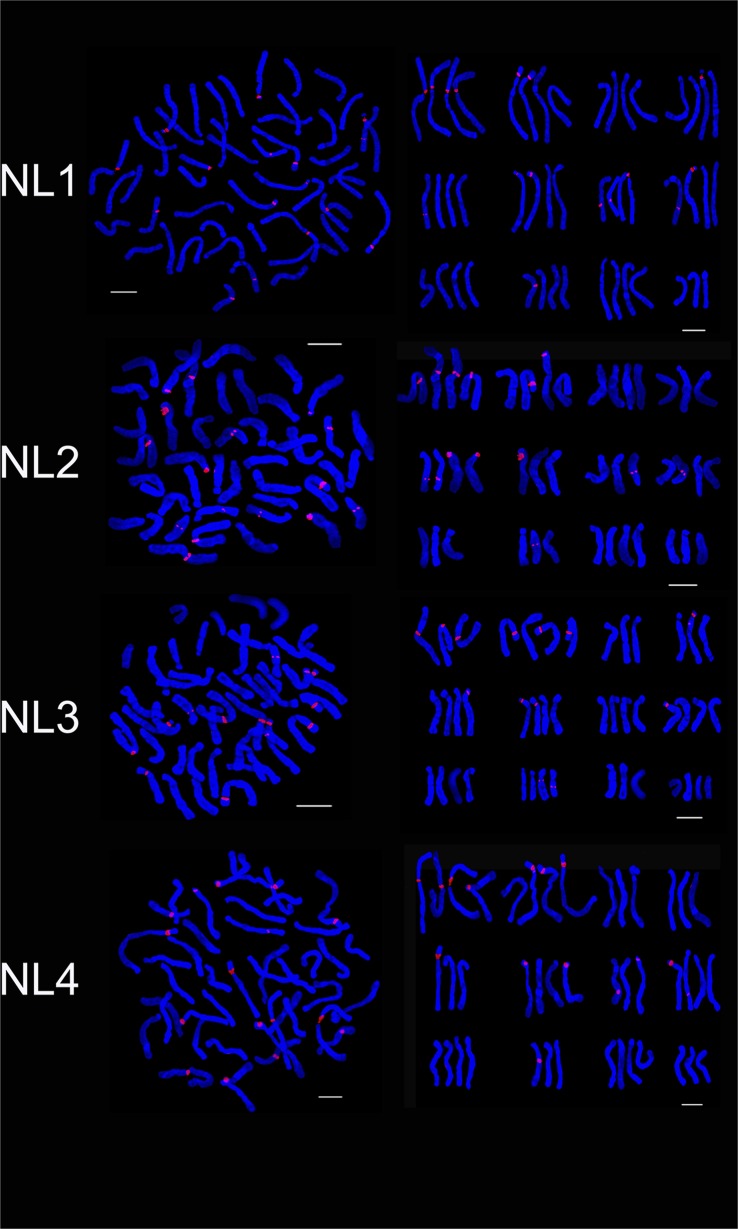
FISH karyotype of the hybrid progenies (NL1, NL2, NL3, NL4) using 45S rDNA probes. NL1, NL2, NL3, NL4 are the progenies of ‘Navona’× ‘Loreto’. Bar = 10 um.

Four hybrid progenies of NL were aneuploid and chromosome number ranged from 42 to 47. The karyotype was 3B. There were not obvious difference between hybrid progeny and the parent in average arm ratio and asymmetrical coefficient (Tables 2 and 3). Although NL2 and NL4 had the same chromosome number, the number and distribution of the 45S rDNA signal loci was different, respectively 14 and 16. NL2 and NL4 respectively had two and three signal loci on chromosome 2 while 1 and 3 signal loci on chromosome 7. There were 2 and 1 signal loci on chromosome 10, NL4 had one signal loci on chromosome 5 but NL2 had none. NL1 had 16 45S rDNA signal loci, two signal loci were identified on chromosome 2, 6 and 8. Three and two signal loci were found on chromosome 1 and 10 of NL3 which were different with other progenies (Figs 4 and 7). The 45S rDNA signal loci were found on chromosomes 1, 2, 3, 6 and 8 of the parents and the progeny (NL).

**Fig 8:**
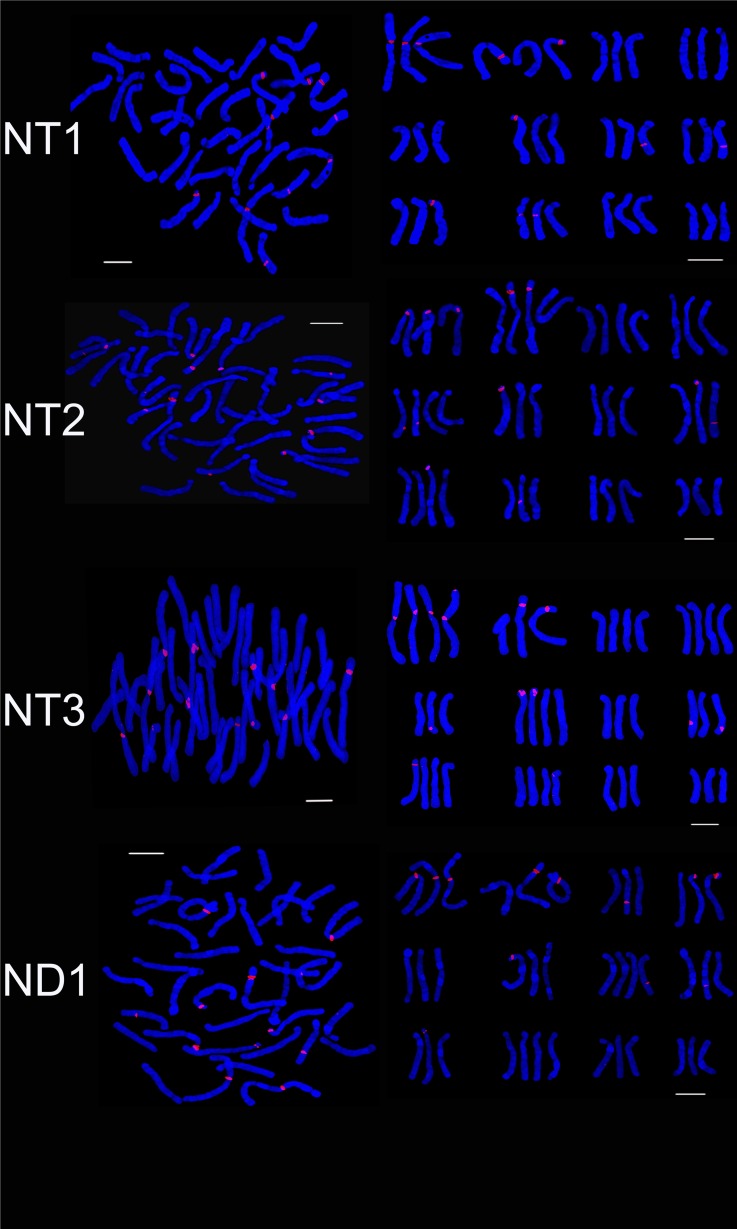
FISH karyotype of the hybrid progenies (NT1, NT2, NT3, ND1) using 45S rDNA probes. NT1, NT2, NT3, NL4 are the progenies of ‘Navona’ ×‘Tresor’; ND1 is the progeny of ‘Navona’ ×‘Detroit’. Bar = 10 um.

The hybrid progeny were triploid or aneuploid and chromosome number was 36 ~ 42 (Table 3). NT2 and NT3 had the same karyotype (3B) with parents while karyotype of NT1 was 3A. NT1 and NT2 had the same chromosome composition with male parent ‘Tresor’. The average arm ratio was close to female parent while the asymmetrical coefficient is similar to the parents. Chromosome number and structure of the hybrid progeny were different. The 45S rDNA signal loci number of NT1, NT2 and NT3 were respectively 11, 12 and 13. On chromosome 1, NT3 had four signal loci while NT1 and NT2 both had three signal loci. One, one and two signal loci were studied on chromosome 6 of NT1, NT2 and NT3. On chromosome 5, NT1 had no signal loci, NT2 had two signal loci and NT3 had one signal loci (Figs 4 and 8). On chromosome 10, NT1 had two signal loci, NT2 and NT3 only had one signal loci in different locations. The 45S rDNA signal loci were detected on chromosomes 1, 2 and 10 of the parents and the progeny (NT).

**Fig 9:**
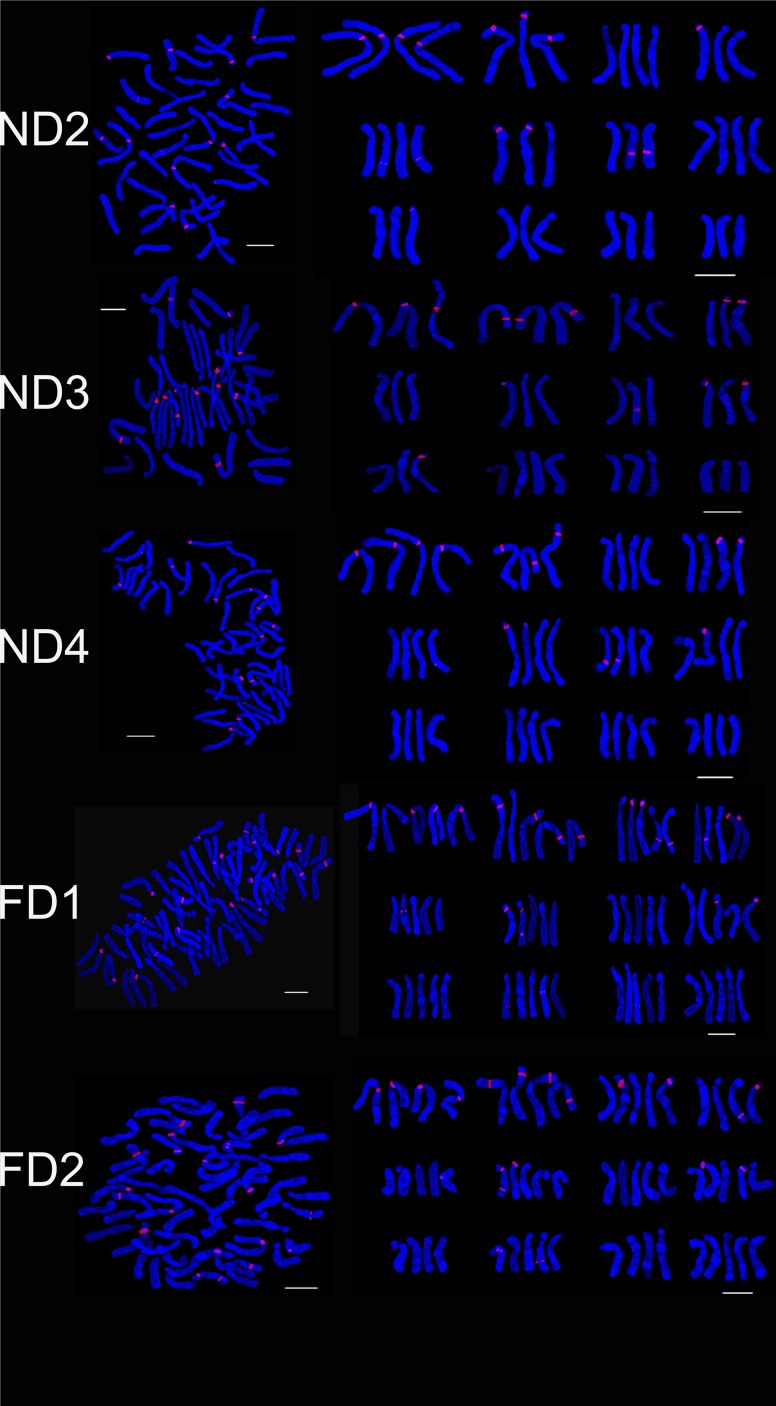
FISH karyotype of the hybrid progenies (ND2, ND3, ND4; FD1, FD2) using 45S rDNA probes. ND2, ND3, ND4 are the progenies of ‘Navona’ ×‘Detroit’; FD1, FD2 are the progenies of ‘Freya’× ‘Detroit’. Bar = 10 um.

**Fig 10:**
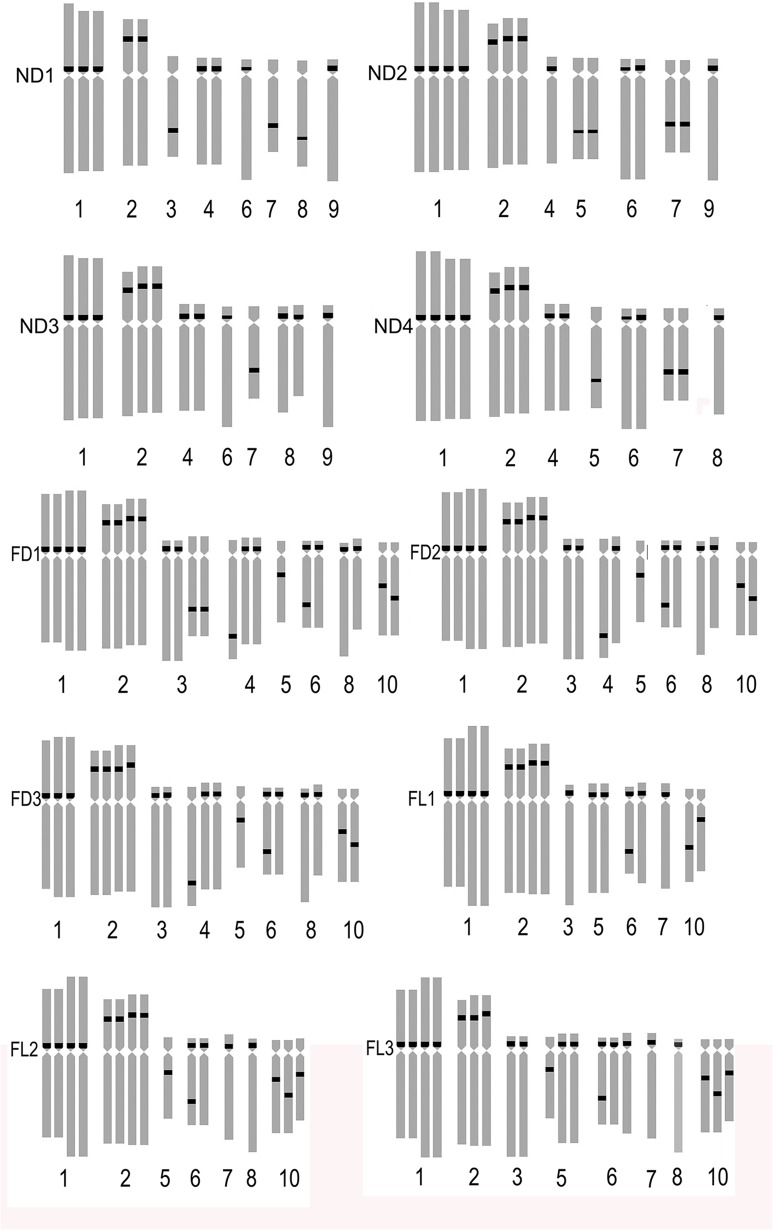
Ideograms showing the distribution of 45S rDNA on the chromosomes in the hybrid progenies (ND, FD, FL). ‘Navona’ ×‘Detroit’, ‘Freya’× ‘Detroit’, ‘Freya’× ‘Loreto’.

Four hybrid progenies were aneuploid, chromosome number varied from 37 to 47. The karyotype type of ND2, ND3 and ND4 was 3B while that of ND1 was 3A. The average arm ratio and asymmetry coefficient were similar to parents (Tables 2 and 3). The number of 45S rDNA signal loci in NT1, NT2 and NT3 was 11, 12 and 13, respectively. On chromosome 1 ND1 and ND3 possessed three signal loci while ND2 and ND4 had four signal loci. On chromosome 2, ND2, ND3 and ND4 had three signal loci while ND1 had two signal loci. On chromosome 6, ND1, and ND3 had two signal loci, and ND2 and ND4 had one signal loci. On chromosome 8, ND1, ND2, ND3 and ND4 had one, zero, two, one signal loci and the signal loci of ND1 located on long arm area. One signal loci were positioned on chromosome 7 of ND1 and ND4 while ND2 and ND4 had two signal loci (Figs 8, 9 and 10). The 45S rDNA signal loci were observed on chromosomes 1, 2, 3 and 6 of the parents and the progeny (ND).

**Fig 11:**
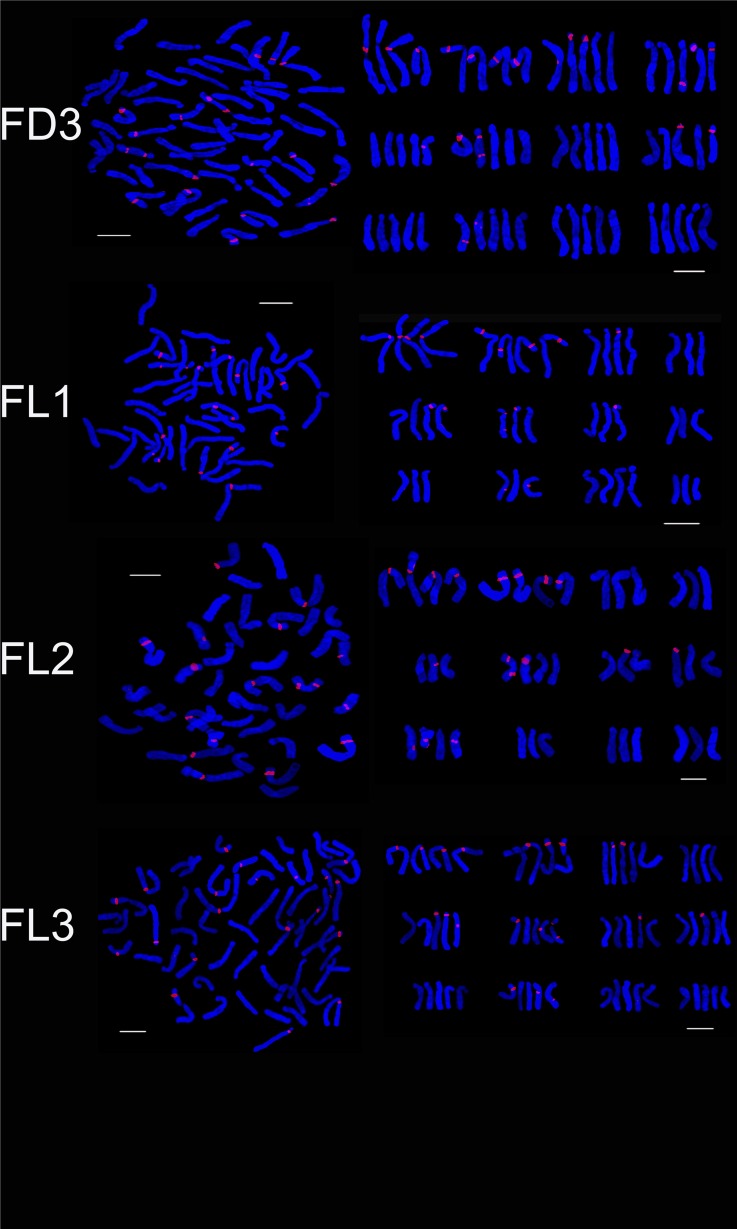
FISH karyotype of the hybrid progenies (FD3; FL1, FL2, FL3) using 45S rDNA probes. FD3 are the progenies of ‘Freya’× ‘Detroit’; FL1, FL2, FL3 are the progenies of ‘Freya’× ‘Loreto’. Bar = 10 um.

The hybrid progeny were pentaploid or aneuploid and chromosome number ranged from 55 to 60. The karyotype type of male parent and three progeny was 3B. The karyotype formula of FD1 was consistent with male parent 'Detroit’ while FD2 and FD3 had the same karyotype formula with female parent ‘Freya. The average arm ratio and asymmetric coefficients were between the parents (Table 3). There was not obvious difference on the number and distribution of 45S rDNA signal loci in hybrid progeny since hybrid progeny had larger number of chromosome and almost inherited the entire chromosome from female parent. FD1, FD2 and FD3 (Figs 9, 10 and 11) respectively had 23, 20 and 20 45S rDNA signal loci. On chromosome 3 of FD1 had four signal loci that two from male parent and two from female parent while FD2 and FD3 just had two from female parent. FD2 and FD3 had the same 45S rDNA signal loci but different distribution. On chromosome 1, FD2 had four signal loci and FD3 had three while FD2 owned two signals and FD3 had three on chromosome 4. The 45S rDNA signal loci were identified on chromosomes 1, 2, 3, 6 and 11 of the parents and the progeny (FD).

The hybrid progeny (FL) was aneuploidy and chromosome number varied from 40 to 57. The karyotype of FL2 was 3A the same as female parent while that of FL1 and FL3 was 3B similar to male parent. FL3 had the same karyotype formula with male parent ‘Loreto’ which were made up of two pairs of metacentrics, a pairs of telocentrics and 9 pairs of subtelocentrics. The chromosome 2 of FL1 was submetacentrics similar to ‘Freya’ while FL2 and FL3 were the same with male parent ‘Detroit’ as metacentrics. There were not obvious difference in average arm ratio and asymmetric coefficients between hybrid progeny and the parents (Tables 2 and 3). The hybrid progeny not only had different chromosome, but also distribution of 45S rDNA signal loci (Figs. 10 and 11). The number of 45S rDNA signal loci in FL1, FL2 and FL3 was 17, 17 and 21, respectively. On chromosome 2, FL1 and FL2 had four signal loci while FL3 only had three signal loci. The number of signal loci on chromosome 3 of FL1, FL2 and FL3 were one, zero and two. On chromosome 5, FL3 had three signal loci while FL1 and FL2 both had two. On chromosome 6, FL3 possessed three signal loci while FL1 and FL2 had two. On chromosome 7 of FL1 and FL3 both owned one signal loci. One signal loci of FL1 and FL3 was located on chromosome 10 where FL2 did not have. The 45S rDNA signal loci were observed on chromosomes 1, 2, 5, 6, 7 and 10 of the parents and the progeny (ND).

### The Application of 2n Gametes in Lily Breeding

The chromosome number of FD1 (‘Freya’ x ‘Detroit’) is 60 (Table 3) according to the analysis of FISH and karyotype. FD1 inherited all chromosomes of female parent (‘Freya’) and half chromosomes of male parent (‘Detroit’) which may be due to the production of the female gamete without meiosis (2n gametes) of female parent. The production of 2n gametes usually occurs in angiosperms and many scholars believe that they are the origin of polyploidy plant species [26, 46]. The 2n gametes are often used in the production of polyploidy before the discovery of colchicines which can doubles chromosomes to restore fertility.

The 2n gametes play an important role in breeding. The allopolyploid by artificial induction could not achieve the restructuring between genomes because of homologous chromosome pairing during meiosis and the heterozygosis was certain while the heterozygosis of sexual polyploidy (2n gametes) with recombination between the parents’ genome in nature was indefinite and increased the genetic variation. Hence, sexual polyploidization has more advantages in the breeding [47]. 2n gametes can also be achieved by restructuring introgression [48, 49] and academics have successfully applied it to breeding [26, 50–52].

Abnormal meiosis is the main source of 2n gametes which can be got during meiotic recombination process in microspore or megaspore [26]. To illustrate the different formation mechanism of 2n gametes, academics have conducted lots of cytology and genetics research [46]. Through genomic in situ hybridization (GISH) and fluorescence in situ hybridization (FISH) analysis, formation mechanisms of 2n gamete in lily hybrid are divided into three types: First Division Restitution (FDR), Second Division Restitution (SDR) and Indeterminate Meiotic Restitution (IMR) [39, 53, 54, 55], which play an important role in creating genetic variation and realizing introgression. Different mechanisms of the hybrids were associated with the genetic relationship of their parents. FDR and IMR mechanism had been confirmed in LA and OA lily hybrids whose parents were distant in genetic relationship. On the contrary, the SDR mechanism was found in *L*. *pumilum* x ‘Enchantment’ hybrid whose parents belonged to Sinomartagon and had close genetic relationship [53].

In the process of breeding, the use of 2n gametes had obvious advantages compared with using somatic autopolyploid. However, the uses of 2n gametes had low production and big randomness and were especially difficult to detect 2n female gamete. So the artificial induction was an important means for increasing the production of 2n gamete. The colchicines and N_2_O are the current reagents to induce the 2n gametes [52, 54].

The methods of hybrid identification mainly include morphology, cytology and molecular markers. The morphological identification of hybrid authenticity is one of the most intuitive ways. However, lily breeding cycle is long and at least 2 ~ 3 years are needed from sowing to flowering. Most scholars apply the cytological and molecular markers to identify authenticity of hybrid and shorten the breeding period. At the molecular level, ISSR, SRAP and RAPD have been widely applied to the hybrid identification of lily [12]. In conclusion, FISH has been effectively used for identifying the authenticity of hybrid and tracking the source of the special chromosome which has the 45S rDNA signal loci in hybrid.

## Supporting Information

S1 Table Chromosome parameters of seven cultivars. (XLS) Asiatic cultivars ‘Renoir’ (R), ‘Gironde’(G), ‘Navona’ (N), ‘Detroit’ (D), ‘Loreto’ (L), ‘Tresor’ (T) and LA cultivar ‘Freya’ (F).
(XLS)

S2 Table The karyotype pattern of seven cultivars. (XLS) Asiatic cultivars ‘Renoir’ (R), ‘Gironde’(G), ‘Navona’ (N), ‘Detroit’ (D), ‘Loreto’ (L), ‘Tresor’ (T) and LA cultivar ‘Freya’ (F).
(XLS)

S3 Table The karyotype characteristics of progenies. (XLS) RG (‘Renoir’ ×‘Gironde’), GR (‘Gironde’× ‘Renoir’), GT (‘Gironde’× ‘Tresor’), LD (‘Loreto’× ‘Detroit’), NL (‘Navona’× ‘Loreto’), NT (‘Navona’× ‘Tresor’), ND (‘Navona’ ×‘Detroit’), FD (‘Freya’× ‘Detroit’), FL (‘Freya’× ‘Loreto’).
(XLS)
